# An epigenetic hypothesis for ovarian cancer prevention by oral contraceptive pill use

**DOI:** 10.1186/s13148-023-01584-9

**Published:** 2023-10-18

**Authors:** Anna S. Avramenko, James M. Flanagan

**Affiliations:** Division of Cancer, Department of Surgery and Cancer, Faculty of Medicine, Imperial College London, 4th Floor IRDB, Hammersmith Campus, Du Cane Road, London, W12 0NN UK

**Keywords:** Ovarian cancer, DNA methylation, Combined oral contraceptive pill, COCP, Prevention

## Abstract

**Background:**

Ovarian cancer is the second most common gynecological cancer type after uterine cancers. In 2020, according to worldwide statistics, there were more than 313,000 new cases of ovarian cancer. Most concerning with ovarian cancer is the poor overall survival, with only 30% of patients surviving for longer than 5 years after diagnosis. The reason for this poor outcome includes late diagnosis due to non-specific symptoms and a lack of any highly effective biomarkers of the early stages of ovarian carcinogenesis. However, it is important to note that some modifiable lifestyle factors can be preventative [pregnancy, breastfeeding and combined oral contraceptives pill (COCP) use].

**Results:**

There is now increasing data reporting the role of epigenetic changes, which are detectable in ovarian cancer tumors, suggesting the possibility that epigenetics may also play a key role in the mechanism of long-term effective prevention of ovarian cancer. To our knowledge, there is a lack of high-quality data on the molecular mechanisms of ovarian cancer prevention, although several hypotheses have been proposed.

**Conclusions:**

This review focusses on the evidence for a proposed novel hypothesis—that COCPs act as a chemoprevention through the impact on the epigenome of the cells of origin of ovarian cancer—fallopian tubes epithelium.

## Introduction

Ovarian cancer is one of the leading causes of death among women worldwide. Ovarian cancer (OC) accounts for an estimated 313,000 new cases and 152,000 deaths worldwide annually [1]. The disease typically presents at a late stage when the 5-year relative survival rate is only 29% and is diagnosed at the early stage quite rarely. Most cases of ovarian cancer are epithelial ovarian lesions, typically one of five histological types (including high-grade serous, low-grade serous, endometrioid, clear cell, and mucinous histotype). There have been several high-profile studies aiming to test an effective prevention program for OC in the asymptomatic population, UK Collaborative Trial of Ovarian Cancer Screening (UKCTOCS) and the prostate, lung, colorectal and ovarian (PLCO) cancer screening trial (PLCO) [2, 3]. The summary from both studies is that screening in this population does not change mortality outcomes for ovarian cancer, and therefore, that screening for earlier detection of ovarian cancer in this population is unwarranted [4]. Therefore, it may prove more fruitful to consider prevention as the best method to reduce ovarian cancer mortality.

While surgical prevention strategies remain the most effective method in high-risk mutation carriers [5], for the general population at average risk levels, at approximately 2% lifetime risk, the most significant preventive effects have been observed after the usage of oral contraceptives. Several studies revealed that the protective effect from contraceptives increases with longer duration of use [6]. A meta-analysis showed that OC incidence was reduced by as much as 50% in oral contraceptive users after 10 years of use [7].

Recent studies have shown that epithelial ovarian cancer (EOC), particularly high-grade serous ovarian carcinomas (HGSOCs) emerge from the tubal epithelium [8]. HGSOC typically remains undiagnosed until advanced stages, when peritoneal dissemination has already occurred. The precancerous landscape in fallopian tubes contains multiple concurrent precursor lesions, including serous tubal intraepithelial carcinoma (STIC), with genetic heterogeneity providing a platform for high-grade serous ovarian carcinoma (a subtype of EOC) evolution [8]. It has become increasingly clear in recent years that many HGSOCs develop from the epithelial precursor lesions on the fallopian tubes rather than from the ovary, which, in humans, is largely devoid of Müllerian epithelium. This new paradigm of ovarian cancer genesis was based on the original observation of dysplastic epithelium in the fallopian tube in women carrying BRCA1 and BRCA2 germline mutations [9, 10]. Several studies compared the effects of oral contraception, tubal ligation, and risk-reducing salpingo-oophorectomy for high-risk groups as risk-reduction strategies [11].

Assuming that EOC’s origin is fallopian tube epithelium and COCPs play a significant role in the chemoprevention of OC, the question arises: what impact does COCP have on the epithelium of the fallopian tubes? The precise mechanism by which OCs have protective effects remains unclear but may be partly due to inhibition of ovulation that may reduce the opportunity for fimbriae to contact the ovarian surface in every menstrual cycle. Ovarian cancer risk reduction accumulates over the time of the exposure to COCPs with the greatest effect observed in women who have used COCPs for more than 10 years [12]. Therefore, the biological mechanism of risk reduction appears to accumulate with increased exposure. It is also clear that the cancer risk reduction due to COCP use is tissue specific. For example, most epidemiological studies have shown that oral contraception is associated with a 1.5–3.3-fold higher relative risk of cervical cancer by promoting human papilloma virus—DNA integration into the host genome, but only in users for > 5 years [13]. There is also a slight increase in the incidence of breast cancer after prolonged intake of COCPs [14–16]. Meanwhile, COCPs exposure is associated with reduced risk of endometrial, ovarian, lymphatic, colorectal and hematopoietic cancers that persists after discontinuation [17]. Therefore, the effects of COCP that reduce risk appear to be tissue specific.

In this review we explore the hypothesis that epigenetic changes caused by COCP in both ovaries and/or fallopian tubes can explain the long-term risk reduction even after short-term exposure from COCP. Epigenetic modifications have been explored in relation to ovarian cancer tumor development [18], accumulated chemoresistance [19] and in response to platinum treatment [20]. Various epigenetic changes have been recognized in OC tissues and their crucial role in development and progression of tumors is now well-established. However, most studies have focused on the epigenetic changes to tumor suppressor or activator genes during tumorigenesis. The question arises: Do epigenetic changes explain the risk reducing effect of combined oral contraceptives in chemoprevention of OC?

## Epidemiological evidence of ovarian cancer risk factors

Approximately 1 in 50 (2%) women in the UK will be diagnosed with ovarian cancer in their lifetime [21]. An individual’s risk of developing cancer depends on many factors, including age, genetics, and exposure to risk factors (including some potentially avoidable lifestyle factors). Mutations of BRCA 1 and BRCA 2 are the most significant non-modifiable risk factors for the incidence of OC and are more strongly associated with HGSC, although occur in other types of OC as well [22].

The evidence for age at menopause as a risk factor is inconsistent. In the European Prospective Investigation into Cancer and Nutrition (EPIC) cohort, age at menopause (> 52 vs. = 45 years) was associated with an increased risk (HR = 1.57, 95% CI 1.16–2.13); however, after women diagnosed with OC within the first 2 years of follow-up were excluded the risk was slightly attenuated (HR = 1.40, 95% CI 0.98–2.00) [23]. A report from the Nurses’ Health Study (NHS) and NHS II found that age of natural menopause was associated with an increased risk of endometrioid tumors (RR = 1.13, 95% CI 1.04–1.22), but not serous invasive (RR) or mucinous tumors (RR) [23].

The association between parity and OC has been studied thoroughly. Pregnancy is an important factor in preventing ovarian cancer. Troisi et al. reported that women who had given birth had a 30–40% lower risk of developing OC than nulliparous women and that the mentioned protective effect increases with each subsequent pregnancy [24]. Adami and colleagues showed that the risk of ovarian cancer decreases by about 10% for each 5-year increment in age at first childbirth (odds ratios 0.89 [0.84–0.94] epithelial cancer, 0.92 [0.77–1.10] stromal cancer, 0.92 [0.65–1.32] germ-cell cancer, 0.93 [0.80–1.09] borderline tumors) [25]. Some other studies calculated the risk of OC decreasing 8% with every pregnancy and another one calculated a decline of 18%, 26%, 33%, and 42% for the first, second, third, and fourth pregnancy, respectively [24, 26].

Breastfeeding is a significant preventative factor for ovarian cancers. A recent meta-analysis revealed a significant protective effect (summary RR = 0.68, 95% CI 0.61–0.76) for breastfeeding that increased with longer duration (summary RR = 0.85, 0.73, and 0.64 for < 6 months, 6–12 months, and > 12 months of total breastfeeding duration) [27]. Thus, lactation protects against EOC, especially with long-term duration [27]. However, prolactin, a hormone that is highly secreted during the breastfeeding period, in some studies was recognized as an inducer of carcinogenesis by regulating gene expression or by activating signalling pathways associated with proliferation and inhibition of apoptosis [28].

COCPs are unquestionably the strongest protective factor and play an important role in preventing ovarian cancer. Substantial reduction in epithelial ovarian cancer risk was observed among women who used OCs for < 1 year if they were recent users (time since first or last OC use within 20 years), each year of OC use provided an average 5% reduction in the odds ratio (OR 0.95; CI 0.92–0.98) [29]. The greatest reduction in risk was observed in women who started OC use before age 20 years and stopped after age 30 years [29]. Most studies confirmed that protective effect of COCPs increases with the longer duration of use (about 20% decrease in risk for each 5 years) that, interestingly, persists for the decades after cessation. Interestingly, the reduction in risk waned among short-term users (< 1 year), who stopped using COCPs 20 or more years before the reference date [29]. However, despite all this epidemiological evidence, it is still unclear what the molecular mechanism are driving this reduction in ovarian cancer risk.

## Previously proposed mechanisms

The origin and pathogenesis of epithelial ovarian cancer has perplexed investigators for decades.

1. Originally, OC was considered to emerge from ovarian tissue. The *‘*incessant ovulation’ hypothesis suggested that HGSOC developed because of repetitive injury to the ovarian surface epithelium (OSE) with each ovulatory cycle. It posits that the number of ovulatory cycles increases the rate of cellular division associated with the repair of the surface epithelium after each ovulation, thereby increasing spontaneous mutations leading to DNA damage produced by oxidative stress [30, 31]. Therefore, early menarche, late menopause, and nulliparity, all of which have more ovulation episodes, increase the risk of developing ovarian cancer.
2. An alternate hypothesis proposes that tumors with a Müllerian phenotype (serous, endometrioid and clear cell) are derived from Müllerian-type tissue and not mesothelium [32]. This Müllerian-type tissue (columnar epithelium, often ciliated) lines cysts located in paratubal and paraovarian locations that have been referred to collectively as the “secondary Müllerian system” [33]. According to this theory, ovarian tumors develop from these cysts. As the tumor enlarges, it compresses and eventually obliterates ovarian tissue resulting in an adnexal tumor that appears to have arisen in the ovary.
3. An HGSOC precursor has been identified in the fallopian tube, especially in fimbriated-end STIC lesions, and although original debated [34], more recent data support this idea. The hypothesis suggests that tumor cells from STIC lesions exfoliate from the fimbriae, and then implant and develop on the ovary [35]. In support of that hypothesis, DNA sequencing revealed that the majority of STIC lesions harbor the same TP53 mutation as the concurrent HGSOC, indicative of their clonal nature. The current notion that high-grade ovarian carcinomas are derived from fallopian tube epithelium precursors is also supported by their highly conserved methylomes, with only a minority of them clustering with ovarian surface epithelium [36].
4. One of the hypotheses has suggested that an inflammatory microenvironment such as cell damage, oxidative stress, and elevations of cytokines and prostaglandins, which happens during the process of ovulation, could mediate mutagenesis [37]. The inflammation hypothesis was supported by the evidence of increased incidence of ovarian cancer among individuals with pelvic inflammatory disease along with the interesting possibility that hysterectomy and tubal ligation could prevent ovarian cancer. Endometriosis, which causes a marked local inflammatory reaction, has been linked to ovarian cancer, in particular endometrioid and clear cell histotypes. By blocking ovulation, during which a large amount of proinflammatory cytokines are released, COCPs prevent the formation of the consistent inflammatory molecules surrounding of the ovaries and fallopian tubes, therefore, prevent the OC [21].
5. An androgen/progesterone hypothesis is still debatable. It suggests that higher levels of androgens, which are increased in menopausal or obese women, and also detected among women with polycystic ovary syndrome (PCOS), were associated with an increased risk of ovarian cancer [38]. Meanwhile, several studies showed that progesterone can protect against ovarian cancer [39, 40]. As COCPs contain progesterone components as well as estrogens, their chemopreventive effects in terms of OC could act through this way. In support to this theory, a long-acting progestin depot-medroxyprogesterone acetate has shown to be associated with decreased risk of ovarian cancer—35% decreased risk overall (OR 0.65, 95% CI 0.50–0.85) and moreover showed a statistically significant trend of decreasing risk with increasing duration of use (*p* trend < 0.001) [41]. In addition, a study with postmenopausal women on estrogens showed the decreased risk of ovarian cancer by adding progesterone. The increased risk in estrogen therapy users was statistically significantly higher than the increased risk in estrogen-progesterone users (*p* = 0.004) [42].
6. The gonadotropin hypothesis has also been proposed as an underlying mechanism to ovarian cancer and states that excessive levels of gonadotropins, related to the surge occurring during ovulation and the loss of gonadal negative feedback for menopause and premature ovarian failure, may play a role in the development and progression of OC [43, 44]. This theory would also explain the decreased risk of ovarian cancer associated with pregnancy and with COCP use, which results in reduced exposure to gonadotropins due to the steroid feedback on the pituitary. In the 2–3 years after menopause, gonadotropin levels are particularly high, such that concentrations of FSH and LH reach a peak of 10–20 times (50–100 mIU/ml) and 3–4 times (20–50 mIU/ml) the values recorded during the proliferative phase of the menstrual cycle, respectively, and after which there is a gradual but slight decline in both gonadotropins [15, 16]. Thus, in support of the gonadotropin theory, the incidence of ovarian cancer climbs dramatically around the age at which most women reach menopause [45]. COCPs cause suppression of the production of the gonadotropin hormones [46], especially their long-term intake which could lead to suppression of the whole reproductive system up to 6 months after cessation. COCPs ability to reduce gonadotropins for a substantial time creates a consistent homeostasis of hormonal levels that may be a contributing factor to the mechanism of OC prevention.

Taking together, the development of OC is complex and might not have a simple direct answer to the question of its origins. It is also clear that the origins may be different depending on which histological type of ovarian cancer is present [47, 48]. One mechanism for the risk reduction due to COCP that has not been extensively explored yet is the epigenetic hypothesis.

## Epigenetic changes induced by COCP

Epigenetics is normally characterized as a heritable and reversible change in gene expression that is not joined by a change in the DNA sequence. There are three main epigenetic changes: DNA methylation, histone modification (chromatin remodelling), and microRNAs (miRNAs) [10].

Methylation is the process of adding a methyl group to the cytosine nucleotide within a cytosine-phosphate-guanine sequence of DNA, referred to as a CpG site. This can act to silence gene expression in that region of DNA. Epigenetic alterations including DNA methylation play a significant role in cancer, from the silencing of tumor suppressors to the activation of oncogenes and the promotion of metastasis.

Evidence that epigenetic changes play a role in the development of OC are confirmed by numerous studies. In ovarian cancer, like many cancer types, two contrasting epigenetic phenomena have been detected: (1) An overall global decrease in DNA methylation that leads to demethylation of several oncogenes and repetitive elements, and (2) specific CpG island hypermethylation associated with the promoters of tumor suppressor genes that deactivate them.

The suggestion that high-grade serous epithelial OC emerges from STIC lesions means that the most important biomarkers of ovarian cancer should be present in STIC lesions before they move to the ovary. Moyle-Heyrman and colleagues determined that fallopian tube has specific gene targets of the estrogen receptor and demonstrated a tissue-specific response to selective estrogen receptor modulators consistent with antagonistic action [49]. Pisanic and colleagues showed that DNA hypermethylation was detectable in STIC samples and was completely absent in fallopian tube epithelia from healthy women [50]. Relying on DNA methylome analysis, Klinkebiel and others revealed that HGSOC more closely resembles fallopian tube epithelium than ovarian surface epithelium [51]. DNA methylome analysis of patient samples from tumor tissue, fallopian tube epithelium, and ovarian surface epithelium showed that dominant changes in the HGSOC epigenome most directly correspond to methylation patterns in the matched fallopian tube epithelium. Pisanic et al. showed that there are indeed detectable hypermethylation loci at *TUBB6*, *IRX2*, and *c17orf64* promoters that reliably differentiate malignant fallopian tube epithelium from benign tissue [50]. In addition, HGSOC-specific hypermethylation in the same loci was detected in the tissue of fallopian tubes with STIC lesions [50].

These data support the tubal origin of EOC and suggests that epigenetic changes are an important step in carcinogenesis. However, analysis of STIC lesions remains inherently difficult as specimens are often scarce and extracting DNA of sufficient purity and yield for genome-wide epigenetic analysis is often not feasible [50].

Hence, Bartlett and colleagues demonstrated that epigenetic reprogramming in even morphologically normal fimbrial cells of *BRCA1/2* mutation carriers, (removed during risk-reducing surgery), which have a high risk for neoplastic transformation, is highly prevalent [52]. Epithelial OC methylation was compared with normal fallopian tube DNA methylation in some studies, one of them even compared methylation differences between the histotypes. 168 genes were identified with altered gene expression in HGSOC compared to normal fallopian tube, with 11.5% hypermethylated at *BRCA1* [52].

Epigenome alterations also have a crucial role in ovarian cancer tumor progression, chemoresistance and relapse. The increased *MLH1* (*DNA* mismatch repair protein) methylation in plasma samples at relapse after carboplatin/taxane chemotherapy of EOC patients is consistent with in vitro observations in ovarian cell line models that cisplatin and carboplatin select for loss of an apoptotic response and acquisition of drug resistance, which is associated with loss of expression of mismatch repair (MMR) proteins and methylation of *MLH1*. This helps to validate methylation of *MLH1* and loss of DNA MMR as clinically relevant mechanisms of acquired drug resistance in EOC [53]. Another study showed that treatment of two of the resistant cell lines with 5-azacytidine, a known inhibitor of methylation, results in re-expression of *MLH1*. Clonogenic assays demonstrate that the 5-azacytidine treated cells show increased sensitivity to cisplatin [54]. Those data are strongly supportive of the reversibility of epigenetic changes and disclose realistic potential for the epidrugs to become a new era for cancer treatment. Recent work has also shown that epigenetic patterns detected in blood are associated with prognosis at relapse. Changes in DNA methylation in response to platinum-based chemotherapy appeared to predict platinum response [55]. Therefore, epigenetic biomarkers may have an important role in ovarian cancer clinical management.

Oral contraceptives are the source of exogenous hormones (estrogen and progesterone) and their impact on the prevention and development of OC has been explored for years. But with the new emerging data about epigenetic changes, their involvement in the chemoprevention of OC might be better understood if epigenetic changes are considered. DNA methylation is generated by a family of three active DNA methyltransferases (DNMTs) such as DNMT1, DNMT3A and DNMT3B. Many studies have indicated that DNMTs are under the regulation of estrogen and progesterone; therefore, DNA methylation may be influenced directly by oral contraceptives [56].

### Effects of estrogen on DNA methylation

Estrogen is known to influence DNA methylation which coordinates both gene expression profiles of epithelial cells and the architecture of the mammary gland [57].

The Impact of estrogen on epigenetic changes was thoroughly investigated in breast tissues, and activation of *SIRT1* (NAD-dependent histone deacetylase silent information regulator 1, which is required for estrogen-induced breast cancer growth) transcription by 17β-estradiol through ERα was shown in breast cancer cell lines [58]. Several experiments demonstrated that 17β-estradiol alters the mRNA and protein expression of DNMTs, especially exclusively increasing the expression of the DNMT3b, acting presumably through estrogen-receptors α (ER α).

Exposure to estrogen is known to change DNA methylation patterns allowing for increased proliferation [59]. Estrogen-driven proliferation increases DNA synthesis by recruiting cells into the cell cycle, thereby increasing the proportion of cells with nascent, unmethylated DNA strands. Although this would lead to the activation of the methylation machinery for maintaining the faithful replication of methylation patterns, several studies have shown that these hyperplastic changes are paralleled by the global loss of methylation. However, a study of the impact of estrogen onto the breast tissue revealed that after 4 weeks of exposure to estrogen no changes in global methylation were found [59]. Only induction of both DNMT1 and DNMT3a was evidently found. The fact that no significant changes in global methylation were detected during short-term exposure to estrogen (1–4 weeks) suggests that the changes induced by estrogen-driven hyperplasia and DNMT activity are balanced in such a way that no apparent differences in the levels of global DNA methylation are detected.

Another study showed that estradiol treatment exclusively increases DNMT3b expression. Since DNMT3b is considered a de novo methyltransferase, these results also suggest that ERα has a role in the formation of new DNA methylation and alters the initiation of transcription via DNMT-mediated DNA methylation [60]. Lifetime estrogen exposure was also associated with epigenetic changes detected in blood DNA, indicating that the effects of estrogen on DNA methylation may take longer to establish or become detectable [61]. Finally, anti-estrogen therapy alongside epigenetic drugs may be a therapeutic option for estrogen positive ovarian cancers [62].

Based on these data, we speculate that estrogen induced DNA methylation changes may occur in genes that would prevent cancer development over time. As an example, estrogen showed reduction in the growth of liver cancer cells through epigenetic impact on the *TH1* and *TFRC* genes [63]. Several studies confirmed the pro-oncogenic impact of estrogen on the development of breast cancer, whereas Yu-Wei Chang’s study disclosed that a long-term exposure of human breast cancer cells to estrogen enhances the chemotherapeutic efficacy of doxorubicin and cisplatin, potentially through an epigenetic mechanism, as the effects were reversed by epigenetic therapeutics [64].

In summary, significant evidence exists that support an epigenetic consequence of estrogen exposure.

### Effects of progesterone on DNA methylation

In contrast to estrogen, progesterone is less controversial in its role in ovarian cancer development, and several studies indicated its positive effect on preventing ovarian cancer. Progesterone acts in the human body through the progesterone receptors (PRA and PRB) to counteract the effects of estrogen. Both receptors are isoforms derived from the *PGR* gene.

Lima et al. showed that progesterone, through the progesterone receptors, decreases migration and invasion of ovarian cells [65]. Increased parity is associated with lower risk for OC [66], which means that protection is tangentially attributable to high progesterone levels. It has been demonstrated that monkeys treated with the progestin-component of the oral contraceptive (levonorgestrel) have increased apoptosis in the ovarian epithelium cells as compared with controls and ethinyl estradiol-treated monkeys [67]. In addition, in mouse oviduct cell models, progesterone, through progesterone receptors, induces necroptosis in Trp53^−/−^ and in immortalized human p53-defective fimbrial epithelium through the TNF-α/RIPK1/RIPK3/MLKL pathway [67, 68]. McGlorthan et al. showed in their study that progesterone treatment induced apoptosis in the in vitro ovarian cells [69]. Another study showed a significant reduction of endometrioid ovarian cancer cell survival that was observed after progesterone treatment in endometrioid ovarian carcinoma [70]. An immunohistochemical study confirmed that epithelial ovarian cancer cells have greater levels of expression of progesterone-induced blocking factor protein than normal ovarian tissue; thus, it is supporting the theory of progesterone being a “cancer-prevention” agent.

In terms of the progesterone effect on epigenetic changes, Xiong et al. reported that DNMT3B in the nucleus of luminal and glandular epithelial cells of endometrial tissues in the ‘high progesterone’ group was significantly higher than that in the ‘normal progesterone’ group [71]. That could be the reason to hypothesize that progesterone acts as a cancer preventer not only through apoptosis induction but also by altering the epigenome, by silencing genes that are responsible for cancer development.

### The effects of COCP on other epigenetic mechanisms

DNA methylation is not the only epigenetic mechanism involved in carcinogenesis. Histone modifications and chromatin packaging play an important role in genome regulation in cancers [72]. In ovarian cancer specifically, histone marks such as the repressive H3K27me3 mark and active H3K4me3 mark are considered markers of bivalent genes, poised to be silenced during drug resistance in ovarian cancers, particularly regulated by the EZH2 protein complex [73]. EHMT 1/2 histone methyltransferases also appear to control PARP inhibitor resistance in ovarian cancers [74]. Indeed, dual inhibition of both EZH2 and EHMTs are a novel avenue for drug development in ovarian cancer [75]. During early carcinogenesis, histone modifications mark changes in enhancers, promoters and chromatin not only in the cancer cells but also non-cancer cells within the tumor microenvironment [76]. With regards to prevention, only one study has reported that COCP increases active histone modifications of the mineralocorticoid receptor target genes [77]. However, the role COCP might be playing in reducing ovarian cancer risk, via histone modifications, is largely under-studied and warrants further investigation.

## The epigenetic hypothesis

Epigenetic changes are an essential part of the process of developing malignancies, including ovarian cancer. COCPs and their components, estrogen and progesterone, are proven chemopreventive agents in the development of OC based on epidemiological evidence. COCPs show long-term and strong protection against OC after even 3 months of exposure, but their mechanisms are still not clear, although several hypotheses for these have been proposed. We have shown that COCPs and their components, estrogen and progesterone, all act to modify the epigenome. However, whether these epigenetic changes are responsible for the reduction in ovarian cancer risk is unknown.

If epigenetic alterations are one of the steps into the timeline of normal tissue-to-tumor development, we can predict that COCPs may interfere with the epigenetics of tubal and/or ovarian epithelium. This hypothesis also supports why even short-term exposure to COCPs leads to life-long protection against the development of OC. To support the crucial role of the epigenetic alterations in cancer development, a study investigated genome level methylation differences among monozygotic twins with *BRCA1* gene mutations, one with ovarian cancer and one without, and their healthy siblings revealed that the differential methylation of 12 different genes was associated with ovarian cancer [78].

If we have a look at that process reversibly, may COCPs directly cause an opposite effect on the gene expression? To our knowledge, there have been no studies yet, which examined COCPs preventive effect for the OC through the impact on the epithelium of ovaries and/or fallopian tubes.

As with all the proposed hypotheses, there are limitations to consider. Firstly, as mentioned earlier, epigenetic changes are reversible, and they could be changed otherwise over a period of time or under the impact of a number of factors. Epigenetic changes that do alter the ovarian cancer risk pattern could reverse and disappear later, a “hit and run” molecular event. In addition, cancer-preventive changes, that occurred under the impact of COCPs, could be reversed by the other pro-carcinogenic impacts further in life. It is equally possible that epigenetic changes induced by COCP are co-incidental, a “passenger event”, and not directly involved in the mechanism of prevention.

It is important to note that our understanding of epigenome alterations in cancer is at the very early stages, so there are no defined mechanisms that could prove the role of epigenetics in the development of ovarian cancer. Epigenome alteration as a result of COCPs actions may have no correlation with further ovarian cancer development or its prevention.

Another arising issue around defining COCPs as an effective chemoprevention of the ovarian cancer is technical difficulties with obtaining the proper amount of tissue samples from the healthy women. To our knowledge, even if salpingectomy in BRCA1/2 mutation carriers is one of the recommended approaches toward OC prevention, still few women undergo that procedure. Moreover, salpingo-oophorectomy is quiet a rare type of the surgery that is performed in the women of reproductive age, so getting ‘healthy’ human tissue samples of both ovaries or fallopian tubes to examine COCPs impact remains challenging.

## Conclusions and future directions

Classical genetic mutations alone cannot explain all the properties of cancer, and it is now understood that epigenetic abnormalities, in addition to genetic alterations, are involved in tumorigenesis. The emerging importance of epigenetics in tumor initiation and in the regulation of cancer-initiating cells suggests that epigenetically regulated genes may be promising therapeutic targets and biomarkers. Determining the mechanisms of ovarian cancer prevention, induced by COCPs, could develop a realistic opportunity for chemoprevention, which would be applicable to all women, especially those groups, who are at high-risk when offering COCPs. It is important to remember the potential harms in increasing risk of other cancers, such as breast cancer, while taking COCPs. In particular, understanding the epigenetic regulation of oncogenes, or cancer-promoting genes, would be important for the development of epigenetic-based prevention approaches.

The aim of this review was to emphasize the lack of data in the field of the prevention of the ovarian cancer, which can support the hypothesis of the role of epigenetic changes that are emerging under the impact of COCPs. We suggest such studies are warranted, where COCPs play a role as an epigenetic regulator to investigate their exact effects in both fallopian tubes and ovarian epithelium and how they can change DNA methylation patterns. By detecting triggered points in the epithelium methylome, furtherly we can determine how long the COCPs epigenetic effects last and what might reverse their cancer-protective effect.

## Data Availability

Not applicable.

## Abbreviations

COCP: Combined oral contraceptives pill
DNMTs: DNA methyltransferases
EOC: Epithelial ovarian cancer
EPIC: European Prospective Investigation into Cancer and Nutrition
HR: Hazard ratio
HGSOCs: High grade serous ovarian carcinomas
miRNAs: MicroRNAs
MMR: Mismatch repair
NHS: Nurses’ Health Study
NHS II: Nurses’ Health Study II
OSE: Ovarian surface epithelium
OC: Ovarian cancer
PLCO: The prostate, lung, colorectal and ovarian
PLCO: Cancer screening trial
PCOS: Polycystic ovary syndrome
RR: Relative risk
STIC: Serous tubal intraepithelial carcinoma
UKCTOCS: UK Collaborative Trial of Ovarian Cancer Screening
